# Necessity of Local Modification for Deep Learning Algorithms to Predict Diabetic Retinopathy

**DOI:** 10.3390/ijerph19031204

**Published:** 2022-01-21

**Authors:** Ching-Yao Tsai, Chueh-Tan Chen, Guan-An Chen, Chun-Fu Yeh, Chin-Tzu Kuo, Ya-Chuan Hsiao, Hsiao-Yun Hu, I-Lun Tsai, Ching-Hui Wang, Jian-Ren Chen, Su-Chen Huang, Tzu-Chieh Lu, Lin-Chung Woung

**Affiliations:** 1 Department of Ophthalmology, Taipei City Hospital, Taipei 103, Taiwan; dac58@tpech.gov.tw (C.-Y.T.); chuehtan@hotmail.com (C.-T.C.); sbodys@yahoo.com.tw (C.-T.K.); daj20daj20@gmail.com (Y.-C.H.); ilunt@ms49.hinet.net (I-L.T.); B0493@tpech.gov.tw (C.-H.W.); 2 Institute of Public Health, National Yang Ming Chiao Tung University, Taipei 112, Taiwan; A3547@tpech.gov.tw; 3 Department of Business Administration, Fu Jen Catholic University, New Taipei City 242, Taiwan; 4 Institute of Traditional Medicine, National Yang Ming Chiao Tung University, Taipei 112, Taiwan; 5 Smart Medical and Healthcare, Service Systems Technology Center, Industrial Technology Research Institute, Hsinchu County 310, Taiwan; crackereidolon@gmail.com (G.-A.C.); r02429007@ntu.edu.tw (C.-F.Y.); cjr@itri.org.tw (J.-R.C.); schuang@itri.org.tw (S.-C.H.); OrionLu@itri.org.tw (T.-C.L.); 6 College of Medicine, National Yang Ming Chiao Tung University, Taipei 112, Taiwan; 7 Department of Education and Research, Taipei City Hospital, Taipei 106, Taiwan; 8 Department of Health and Welfare, University of Taipei, Taipei 100, Taiwan; 9 Department of Health Care Management, National Taipei University of Nursing and Health Sciences, Taipei 112, Taiwan; 10 Department of Medicine, National Yang Ming Chiao Tung University, Taipei 112, Taiwan

**Keywords:** diabetic retinopathy, deep learning algorithms, model localised, Taiwan, predict

## Abstract

Deep learning (DL) algorithms are used to diagnose diabetic retinopathy (DR). However, most of these algorithms have been trained using global data or data from patients of a single region. Using different model architectures (e.g., Inception-v3, ResNet101, and DenseNet121), we assessed the necessity of modifying the algorithms for universal society screening. We used the open-source dataset from the Kaggle Diabetic Retinopathy Detection competition to develop a model for the detection of DR severity. We used a local dataset from Taipei City Hospital to verify the necessity of model localization and validated the three aforementioned models with local datasets. The experimental results revealed that Inception-v3 outperformed ResNet101 and DenseNet121 with a foreign global dataset, whereas DenseNet121 outperformed Inception-v3 and ResNet101 with the local dataset. The quadratic weighted kappa score (κ) was used to evaluate the model performance. All models had 5–8% higher ***κ*** for the local dataset than for the foreign dataset. Confusion matrix analysis revealed that, compared with the local ophthalmologists’ diagnoses, the severity predicted by the three models was overestimated. Thus, DL algorithms using artificial intelligence based on global data must be locally modified to ensure the applicability of a well-trained model to make diagnoses in clinical environments.

## 1. Introduction

Diabetic retinopathy is one of the leading causes of blindness worldwide. However, there are no specific symptoms of early diabetic retinopathy, which results in both delayed diagnosis and disease progression in diabetic patients. Thus, the popularity of deep learning algorithms predicting vision-threatening diabetic retinopathy is arising. In recent years, deep learning has achieved great success in medical image analysis. However, most works directly employ algorithms based on convolutional neural networks (CNNs), which ignore the fact that the difference among populations is subtle and gradual [1]. Moreover, few reports have mentioned the legitimacy of an algorithm based on a global dataset (internationally developed among various ethnic races) being applied to a local population, especially between different races [2].

State-of-the-art artificial intelligence (AI) techniques, specifically deep learning (DL), have driven the development of AI-powered computer-aided detection (CADe), computer-aided diagnosis (CADx) systems and all kinds of applications [3]. For example, computer-aided detection for DR diagnosis has become a promising tool for the early detection and severity grading of DR due to the great success of deep learning [4]. Most current DR diagnosis systems do not achieve satisfactory performance or interpretability for ophthalmologists due to the lack of training data with consistent and fine-grained annotations. Nonetheless, the breakthrough of DL algorithms in image classification and recognition has helped solve the technical barriers and difficulties encountered in previous research. However, more studies are needed to improve the interpretation of existing AI-powered CADe and CADx systems [2,5].

The application of machine learning in CADe systems is often limited by feature expression methods and the number of extractable images. However, owing to the development of DL and the improved computing capabilities of high-end graphics cards, high-level feature expression capacities can be attained. For example, DL is being used in the detection and diagnosis of cancer [6,7]. Owing to the urgent need to prevent blindness in populations—which cannot be achieved using the current technologies and medical personnel—ophthalmology accounts for plenty of AI research regarding tailoring telemedicine to large-scale medical screening programs [8]. For instance, the authors of one study developed an AI-based system to automatically identify patients with referable diabetic retinopathy (DR); their system exhibited higher sensitivity (96.8%) and specificity (87.0%) than other systems based on conventional machine learning algorithms [9]. Similarly, the authors of another study trained an AI-based algorithm for DR on a larger dataset (N = 128,175), achieving a performance only comparable to that of seasoned ophthalmologists (sensitivity: 97.5%, specificity: 93.4%) [10]. Both studies can serve as a basis for the development of a large-scale DR screening program. In addition to DR, DL techniques have been applied in systems for detecting glaucoma and age-related macular degeneration [10,11,12]. These systems, which are developed to improve telemedicine in ophthalmology, have been trained on tens of thousands of fundus images and have demonstrated screening performance similar to that of retinal specialists. However, most of the training data for these AI systems have been collected from patients of a single region, ethnicity, or culture. This inevitably leads to the problem of whether an AI system can make acceptable medical assessments on incoming data, which may have distinct features from those in the data used to train it. Against this backdrop, we set to investigate the necessity of local modification for the DL model in predicting the severity of DR. “Local modification” is similar to the term “globalization”. To this end, we built sets of interpretation models of the severity of DR through DL network frameworks at various depths. In contrast to the image composition of the training and validation sets used in the model training phase, we used local data as the test dataset to re-evaluate the model performances and to explore the necessity of local modification of the model through accuracy, sensitivity, and specificity.

Considering the aforementioned applications in ophthalmology, AI-based screening systems for DR that use fundus images were the first to be considered ready for commercialization. According to systematic reviews, DR is among the six most common causes of blindness worldwide and is considered the main cause of blindness in the working-age population [13,14]. In addition, a study on healthcare costs in patients with DR demonstrated that the costs of treating patients with progressive DR were twice that of patients in stable conditions [13]. Therefore, the routine screening of patients with DR is crucial for reducing healthcare costs and increasing the quality of life of patients with DR. IDx-DR is a notable AI system that addresses this challenge. IDx-DR completed its clinical trial with 900 patients with diabetes (sensitivity: 97.4%, specificity: 89.5%) and was approved by the US Food and Drug Administration in 2018. In addition, Verily and Google launched a DR screening tool in India, particularly in remote and rural communities where few patients with diabetes have access to routine care. These deployments and clinical trials have indicated that commercialised AI-based screening systems will be available soon. However, because these systems for screening DR have been developed for use in different geographical regions, their performance on incoming fundus images should be investigated. In particular, the factors that ophthalmologists or medical professionals consider when interpreting reports from an AI for DR screening, especially those from images not trained on the local DR dataset, should be investigated.

Regional and ethnic factors that contribute to the variability in fundus images should be considered before an AI-based DR screening system is deployed. Although lesions related to DR may be similar among patients of all ethnicities, ethnic differences in the background pigmentation of the fundus and optical structures can influence the performance of an AI screening system [4]. For instance, high myopia is more prevalent among Eastern Asian populations than Western populations [15]. Therefore, darker pigmentation in the fundus makes the evaluation of the severity of red lesions, including microaneurysms and intrarenal haemorrhages, difficult for an AI trained on fundus images mainly from white American patients, leading to more false positives. Moreover, structural changes caused by myopia in the fundus, such as myopic conus and tigroid appearance, may confound an AI system’s reading in different ethnic fundus backgrounds. An AI system may identify those structural changes as diabetic lesions if such features are uncommon in the training dataset. The ability of a single AI system to be applied to various ethnic races remains an issue, and it should be carefully investigated and inspected.

**Figure 1:**
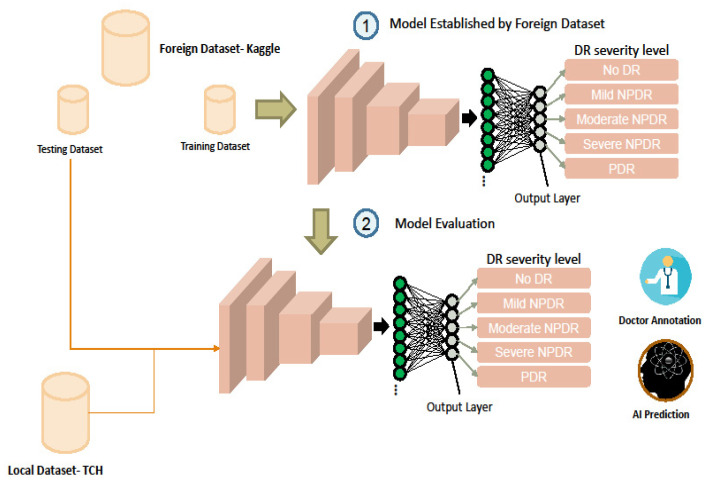
Overview of this study. There are two phases. Phase 1: Model established by the foreign dataset. Three different DL network architectures were trained from the open dataset. The influence of the model architecture on the difference in the interpretation is excluded. Phase 2: Model evaluation. There were two testing datasets used for model evaluation. The test dataset in the foreign dataset was used for comparison with other papers. It was used to ensure that three models have a certain degree of interpretation accuracy. The local dataset was used to test whether the model has a different interpretation accuracy between the two datasets.

We used different training and learning model architectures (three different DL network architectures) to eliminate the possible interference of network characteristics. It is more objective to explore the effects of training and testing datasets from different groups on the interpretation of DR. Retrospective data from community hospitals also contained more localised characteristics. The local dataset used as the test dataset was obtained from local community hospitals. All samples from the dataset were labelled as joint diagnoses by at least two or more experts. The investigation flowchart is shown in Figure 1. To deploy an AI-based DR screening system in clinical practice in Taiwan, we investigated the performance of a well-trained DL model for DR screening based on a worldwide open dataset and a local dataset. The local dataset was collected retrospectively from a local hospital in Taiwan. Furthermore, suggestions for AI system deployment in medical practice and how AI algorithms can be improved are presented.

## 2. Materials and Methods

### 2.1. Dataset

**Figure 2:**
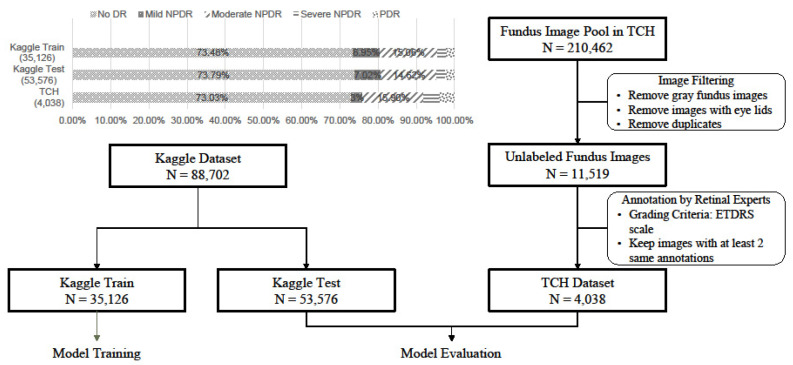
Distribution of disease severity between the two datasets. We trained the 3 models from the Kaggle Train (N = 35,126) dataset. The Kaggle Test (N = 53,576) and TCH (N = 4038) datasets were used for model evaluation.

In this study, a large public dataset from the Kaggle Diabetic Retinopathy Detection competition was used. This dataset is available on eye PACS (available online: https://www.kaggle.com/c/diabetic-retinopathy-detection, accessed on: 20 December 2016), a free platform for retinopathy screening in the United States. Because of this large dataset, there are various symptom samples. Therefore, in this study, we regarded this dataset as a global dataset. The overall data collection process is illustrated in Figure 2. The dataset consisted of 88,702 images, each of which was paired with one of the five severity levels of DR under the Early Treatment Diabetic Retinopathy Study (ETDRS) scale, namely no DR, mild non-proliferative DR (NPDR), moderate NPDR, severe NPDR, and proliferative DR (PDR). These images were further sorted into a training dataset (35,126 images) and a testing dataset (53,576 images), which were named the Kaggle Train and Kaggle Test datasets, respectively. Since the data were collected from primary care institutions, there were serious data imbalances in samples of different severities. The number of patients without DR was much higher than that of patients with PDR. In the model training stage, data augmentation is applied to solve the above problems. The class distributions between the training and testing datasets are similar. Only the Kaggle Train dataset was used to train the DL model for DR detection. Model performance was also evaluated based on the Kaggle test dataset.

The local Taiwanese dataset was comprised of 4038 fundus images, and the prevalence rates of no DR, moderate NPDR, severe NPDR, and PDR were 73.03%, 3.00%, 15.90%, 4.09%, and 3.99%, respectively. All images were retrospectively collected from patients with diabetes who visited the ophthalmology department of Taipei City Hospital (TCH) between 1 January 2007 and 31 December 2017. Two retinal specialists independently interpreted each image. The ETDRS scale was used as the grading criteria for the local dataset, as well as the Kaggle dataset. If inconsistency in the two specialists’ annotations was observed, a third retinal specialist made the third annotation. A total of 18 ophthalmologists participated in the annotation process. In this study, the local dataset was named the TCH dataset and was only used to evaluate the applicability of the DL model, which was trained on the Kaggle Train dataset, to a Taiwanese population. Before training and testing, all fundus images were resized and padded to a pixel resolution of 512 × 512 pixels. Padding with zeros was performed to retain the aspect ratios of the original fundus images.

### 2.2. Model Architecture

Under limited training data, transfer learning is an important tool in machine learning. It tries to transfer the knowledge from the source domain to the target domain by relaxing the assumption that the training data and the test data must be identically distributed. To fully investigate the applicability of DL models trained on the Kaggle Train dataset for DR detection, three champion convolutional neural network (CNN) architectures from the ImageNet Large Scale Visual Recognition Challenge (ILISVRC) were used in this study: Inception-v3, ResNet101, and DenseNet121 [13,14,15]. The factorization process was an important design proposed in the Inception v3 model. It can speed up the operation by reducing the model parameters and increase the nonlinear variation of the model. With the research trend of deepening the model network, gradient dissipation has become a major problem in model training. ResNet utilizes a residual structure, which can not only effectively improve the network depth, but also greatly reduce the possibility of gradient dissipation. In the face of the gradient dissipation problem caused by the deepening of the model layer, DenseNet adopts a more radical structure (channel-wise concatenation). It uses the features of different layers as a reference for each layer of the network, realizes the reuse of features and greatly reduces the computational load of the model. Multiple models were used to verify that this phenomenon was not limited by the characteristics and depth of the selected model. Inception-v3 is characterized by various kernel sizes in the network designed to learn features with distinctive sizes and shapes. However, ResNet101 and DenseNet121 feature residual connections between layers in the network. This feature enables these two networks to have a much deeper network structure than Inception-v3 and mathematically guarantees that redundant features are not learned.

### 2.3. Model Training and Testing

**Figure 3:**
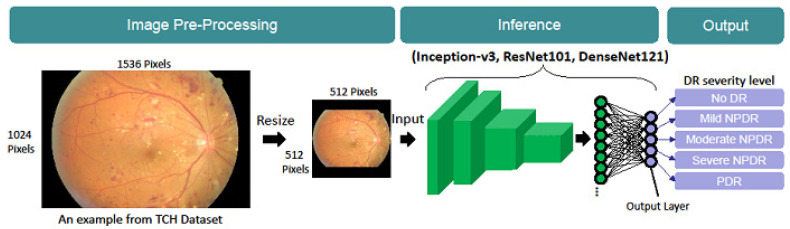
Pipeline from an original fundus image to the DR severity level prediction.

It is not easy to obtain abundant and complete training annotation data, especially in professional fields such as medical treatment and robot vision. Researchers use transfer learning to accelerate model training convergence. In addition, in the pre-training stage, the researchers also increased the variability of the existing data sets by means of data augmentation, including brightness, saturation, rotation, cropping and contrast limited adaptive histogram equalization. It also improved the data imbalance problem. Three pre-trained models (i.e., Inception-v3, ResNet101, and DenseNet121), which were previously trained on the ImageNet dataset, were restored as the base network for fine-tuning. Subsequently, the output layers in these three models were truncated to produce only five values. Each value corresponds to one of the five DR severity levels. The pipeline from the original fundus image to the generation of the five DR severity levels is shown in Figure 3.

During training, batches of fundus images were randomly sampled from the Kaggle Train dataset. After the batches were formed, image augmentation was performed to randomly rotate the fundus images and jitter their color and brightness. This helped in model generalization for the untrained dataset. The batches were then passed to the model to obtain severity level predictions. In addition, 5-fold cross-validation was used for model training and validation. Samples of different severity levels were evenly distributed five times. In each stage of training, there were four folds for the model to modify the network parameters, and the other was used for verification. The above operations are repeated to reduce the interference of the data imbalance on the model training results. Finally, the differences between predictions and annotations were calculated, and an optimization step was performed until model convergence was achieved. Both the Kaggle Test and TCH datasets were used as the testing dataset to evaluate the model performance.

### 2.4. Evaluation Metrics

The evaluation metrics for model performance in DR detection included five-class accuracy (ACC), sensitivity (SEN), precision (PRE), and quadratic weighted kappa score (κ). These four metrics are described in Equations (1)–(4). In the calculation of ACC, SEN, and PRE, $TP_{class}\;$represents the number of accurate predictions of a specific class by the model. For instance, $TP_{1}$ represents the number of true predictions of no DR by the model (where 1, 2, 3, 4, and 5 denote no DR, mild NPDR, moderate NPDR, severe NPDR, and PDR, respectively). $N_{all}\;$represents the total number of images in the dataset, and $N_{class\;}$indicates the number of images of a specific class ranging from 1 (no DR) to 5 (PDR). Predicated $N_{class}$ refers to the number of images in which the model is classified into a specific class. κ was calculated using the scores of the two raters. In this case, one score was obtained from the ophthalmologists, and the other score was obtained from the model predictions. The κ index was used in the evaluation of the classification results to reduce the influence of data imbalance. κ was calculated as follows. First, an *N* × *N* histogram matrix $H_{ij}$, each element of which corresponded to the number of samples assigned a rating i by the ophthalmologists and a rating j by the model, was developed. Second, an *N* × *N* histogram matrix of the expected ratings $M_{ij}$ was established. Each element in $M_{ij}$ indicates the expected probability that the ophthalmologists would assign rating i and the model assigns rating j to an image. This was calculated as the outer product of each rater’s histogram vector of ratings. Both $H_{ij\;}$and $M_{ij}$ were normalised to have identical sums. Finally, the class weight matrix $W_{ij}$ was established to represent the agreement between the ophthalmologists and the model. The elements along the diagonal are zero, whereas those off the diagonal represent the degree of disagreement (square of the difference between i and j). All values were normalised using a maximum squared difference of 16. Therefore, κ was derived from a combination of three matrices (i.e., $X_{ij}$, $M_{ij}$, and $W_{ij}$).

(1) $$ACC=\frac{TP_{1}+TP_{2}+TP_{3}+TP_{4}+TP_{5}}{N_{all}}$$

(2) $$SE=\frac{TP_{class}}{N_{class}}$$

(3) $$PRE=\frac{TP_{class}}{Predicated\;N_{class}}$$

(4) $$\kappa =1-\frac{\sum_{i=1}^{k}\sum_{j=1}^{k}W_{ij}H_{ij}}{\sum_{i=1}^{k}\sum_{j=1}^{k}W_{ij}M_{ij}}$$

Confusion matrices were established to compare the ophthalmologists’ annotations and model predictions on two testing datasets (Kaggle Test and TCH). Moreover, an overestimation rate was calculated to compare the performances of the models on these datasets. The overestimation rate was calculated as the percentage of images in a class that the model misclassified as being more severe than the ophthalmologists’ ratings. For instance, if an image is annotated by ophthalmologists as exhibiting mild NPDR but classified by the model as exhibiting moderate NPDR, severe NPDR, or PDR, it is regarded as an overestimated image.

### 2.5. Implementation

During the training of each model (Inception-v3, ResNet101, and DenseNet121), the following hyperparameters were set to be as similar as possible: the learning rate was 0.0005, the number of epochs was 200, the dropout rate was 0.2, and optimisation was performed using Adam, where beta1 was set to 0.9, and beta2 was set to 0.999. Because the model sizes of ResNet101 and DenseNet121 were larger than those of Inception-v3, the batch sizes for ResNet101 and DenseNet121 were set to 36 samples/epoch, whereas for Inception-v3, this value was set to 48. Regarding the loss function, the multiclass cross-entropy loss and the loss calculated using κ were used.

During model training, validation was performed using the validation dataset. If the new verification result was higher than the previous result, the model would be saved. The model was continuously trained and verified until the performance of the model did not improve. The final recorded model was verified using a local TCH dataset.

## 3. Results

This study demonstrated the necessity of localised modification when a well-trained model was deployed in foreign countries. The foreign Kaggle dataset was used to train the AI model with three types of CNN architecture, and the local TCH dataset was used to validate the trained model.

Although this study only selects three common models through transfer learning to explore whether the model is necessary in the local language, the findings are still very interesting. In theory, the deeper the layers of the model, the more abundant and diverse the extracted features, and the better the performance of the model. The performance of the ResNet model with the largest parameters is not as good as that of Inception v3. In the model performance of the TCH Test dataset, the DenseNet model with the smallest parameters outperformed both.

First, we evaluated the performance of the models trained using the Kaggle Train dataset on both the Kaggle Test and TCH datasets to investigate the effects of model architecture on the ability of the model to detect DR in Taiwanese patients with diabetes. For comparison, the ACC κ and overestimation rates of each model for both datasets were calculated. To objectively discuss the influence of the trained model when applied to a foreign region, we used three types of CNN architectures with the same training dataset to train the AI model for DR detection. The ACC indexes of the three architectural models using the foreign and local datasets were approximately equal.

Second, when we accounted for differences among categories (for 5 levels of DR severity), κ for the TCH dataset (Inception-v3: 85.32%, ResNet101: 83.60%, and DenseNet121: 85.96%) were higher than those in the Kaggle Test dataset (Inception-v3: 79.33%, ResNet101: 78.12%, and DenseNet121: 77.21%), regardless of the model architecture, as shown in Table 1.

In the model verification stage, DenseNet121 under the local data set (TCH) verification was better than the foreign data set (Kaggle) in the model interpretation. The data are presented in Table 1.

Regarding both accuracy or quadratic kappa, DenseNet performed better in the local dataset. In contrast, Inception-v3 and ResNet101 showed opposite results to DenseNet121 in the verification phase. In the foreign datasets, the two models had better interpretation performance than the local datasets. Because the prediction of DR severity was a multicategory task, we used two indexes, namely SEN and PRE, to evaluate the performance of these models. Among the three CNNs, Inception-v3 produced a higher weighted average precision (85.23%) and an 83.80% recall rate. This model architecture may be optimal for clinical applications. Moreover, (1) for the three model performances in each severity classification, under the same model architecture, the SEN and PRE of the foreign and local data sets are generally similar for each severity. In particular, mild NPDR (class 1) in the Kaggle Test dataset differed considerably from those in the TCH dataset. (2) If the model is to be used for early screening, the two-category indicators (i.e., referral and non-referral) can provide medical staff with a clearer diagnosis. Only two categories are required: referral and non-referral. We can easily use the classifier, No DR, to select all normal and abnormal patients out. The performance of No DR is much better than that of the foreign dataset (both SEN and PRE are approximately 95%), as shown in Table 2.

The overestimation rates of each class for the TCH dataset were higher than those for the Kaggle test dataset. This suggested that the model trained with the Kaggle Train dataset was inclined to make predictions of more severe DR than the ophthalmologists. The overestimation rates are summarised in Table 3.

## 4. Discussion

The key to the quality of the model depends on the dataset. In theory, the richer and more uniform the dataset, the higher its representativeness and versatility. However, it is not easy to collect datasets, especially medical imaging datasets, because they involve patient privacy. The foreign dataset and the Kaggle dataset used in this study contain nearly 100,000 pieces of data. The data cover a wealth of symptoms of DR. Even so, we noticed a data imbalance between different severity levels.

DL algorithms are useful for disease diagnosis based on medical images, particularly in ophthalmology. Moreover, most datasets collected for training and testing DL algorithms are obtained from single sources (e.g., a hospital or organization in a single country). Applying DL algorithms to new data possibly possessing features that differ from single sources may disperse the results. In this study, we investigated the applicability of well-trained DL models for DR screening to a local Taiwanese dataset, as well as the effect of the model architecture on such applicability. The study results provide references and evidence for the development of DL algorithms in ophthalmology and the introduction of such systems in clinical practice.

In our study, ***κ*** for the TCH dataset was generally 5–8% higher than for the Kaggle test dataset. The equation for κ penalizes misclassified and extreme predictions more heavily than for other predictions; therefore, more PDR images in the TCH dataset may have been correctly detected by the model trained on the Kaggle Train dataset. This is supported by the higher SEN for PDR images in the TCH dataset than in the Kaggle test dataset. However, although the SEN for PDR images was high, the PRE was generally lower in the TCH dataset. The feature space for PDR learned using our trained models with the Kaggle Train dataset was larger than the actual feature space for PDR in the TCH dataset. Consequently, the images with no DR or NPDR in the TCH dataset were prone to misclassification as PDR in this model.

Furthermore, the higher overestimation rates for each class (no DR, mild, moderate, and severe) in the TCH dataset for all model architecture types suggest an inherent difference between the Kaggle and TCH datasets. This difference may be attributable to regional and ethnic factors. Specifically, because myopia prevalence is the highest in Asia [16], the average eyeball axial length in the Taiwanese population is generally large. This causes striped or spotted appearances, called tigroid patterns, in fundus images [17]. These patterns may be mistaken for red spots, abnormal vessel deformations, or even exudates, which are common fundus lesions in DR. Overestimation rates were high because all three models were trained on the Kaggle Train dataset rather than on the TCH dataset. Unlike our experiment, Ting et al. trained a DL model for DR with fundus images (n = 76,370) obtained from 10 multiethnic cohorts with diabetes [18]. The evaluation of their model with 112,648 images demonstrated consistent results with the retina images from patients of different races and ethnicities (and therefore with varying fundi pigmentation). Although their model was robust in representing ethnic differences in pigmentation, whether such a model could discriminate tigroid patterns from vessel abnormalities remains uncertain. Apart from pigmentation, various patterns similar to abnormalities in fundus images with DR should be further investigated to ensure that the model is not affected by ethnic differences.

Two aspects should be considered if a model trained on a large-scale dataset is introduced in clinical practice. First, certain metrics, such as the overestimation rate, should be used to determine whether such a model requires fine-tuning. This can be addressed using incremental learning, which measures concept drift and fine-tunes the model if necessary [19]. Second, ophthalmologists who work with such an AI model should note that images obtained from patients with myopia are more likely to be misclassified. Image classification can be confirmed through manual investigation of the regions of interest (ROIs) or class activation maps [20] that the AI model is used on to determine DR severity in fundus images. For example, if an image is classified as NPDR or DR by the AI model and features caused by myopia appear in ROIs, the ophthalmologist can treat the image as misclassified and ignore the model’s prediction. In the future, user interface design for AI-empowered CADe and CADx systems should consider a process for easily detecting misclassified images to reduce the workload of ophthalmologists.

To select the optimal model architecture for the AI-empowered DR prediction system, three models were included in the present study. Identification of the optimal model architecture based on the ACC and overestimation rates is difficult. However, when model complexity is considered, Inception-v3 may be the optimal model among the three. Because ResNet101 and DenseNet121 models are more complex than Inception-v3, they theoretically should outperform Inception-v3. However, no significant improvement was observed when ResNet101 and DenseNet121 were used; the model complexity of Inception-v3 was sufficient for DR detection in the fundus images.

## 5. Conclusions

The innovation of big data and AI has not only changed human lives but also promoted advancements in medical technology. The popularity of DL algorithms for predicting vision-threatening DR is increasing. However, only a few reports have investigated the legitimacy of an algorithm based on a global dataset applied to a local population, especially between different ethnic races.

In this study, we used a global DR database from Kaggle as the training and validation database for AI-based detection of DR. To ensure adequate performance, the local TCH dataset was used for model verification. The local DR database, TCH, was used to simulate the results of the model deployment to a different location. The experiment showed that the AI model performed well in two categories: referral and non-referral. However, determining the severity of DR using AI is challenging. By analyzing differences in ***κ*** and overestimation rates, this study demonstrated that ethnic differences should be considered when a DL model is trained for DR screening. Regardless of the selected model structure, the differences between the sample and the population between the datasets are not easy to eliminate. Therefore, the researchers suggest that after the model training has come to an end, another batch of local data samples can be prepared. Compared with the training method of transfer learning, the existing model parameters are optimized. In this way, the applicability of the model can be greatly improved. Especially in the medical field, it is not easy to obtain a large amount of training and verification data marked by many experts.

To the best of our knowledge, our study showed that model-localized modification plays a vital role in the application of DL algorithms to medical images. For example, DL models for DR detection in clinical practice should first be locally modified to learn region-specific features. This will prevent the model from referring to patients without significant fundus abnormalities. The introduction of information and communication technology should improve existing medical conditions and avoid derivative medical problems.

## Figures and Tables

**Table 1:** Model performance with the three types of CNN architecture.

Model	Dataset	Accuracy (%)	Quadratic Kappa (%)	Weighted Average Recall/Precision (%)
Inception-v3	Kaggle Test	84.64	79.33	84.64/82.41
	TCH Test	83.80	85.32	83.80/85.23
ResNet101	Kaggle Test	83.89	78.12	83.89/81.47
	TCH Test	82.99	83.60	82.99/84.91
DenseNet121	Kaggle Test	84.05	77.21	84.05/81.29
	TCH Test	84.67	85.96	84.67/84.80

**Table 2:** SEN and PRE with the Kaggle test and TCH datasets.

Model	Metrics	Dataset	No DR (%)	NPDR (%)			PDR (%)	Weighted Average (%)
				Mild	Moderate	Severe		
Inception-v3	SEN	Kaggle	95.86	18.15	66.75	40.02	58.07	84.64
		TCH	94.34	6.61	59.66	40.61	89.4	83.80
	PRE	Kaggle	89.58	47.96	70.15	49.24	71.01	82.41
		TCH	97.10	27.59	62.99	50.00	35.82	85.23
ResNet101	SEN	Kaggle	97.46	16.67	59.39	40.12	58.28	83.89
		TCH	94.64	19.83	55.76	23.64	86.34	82.99
	PRE	Kaggle	88.55	47.34	71.94	44.14	58.52	81.47
		TCH	96.31	28.24	68.32	38.61	32.33	84.91
DenseNet121	SEN	Kaggle	98.62	9.30	60.41	29.79	54.45	84.05
		TCH	97.12	1.65	59.50	20.00	85.71	84.67
	PRE	Kaggle	87.30	53.50	72.76	44.09	66.84	81.29
		TCH	95.79	28.57	70.35	35.48	34.07	84.80

**Table 3:** Overestimation rates of Inception-v3, ResNet101, and DenseNet121.

Model	Dataset	No (%)	Mild (%)	Moderate (%)	Severe (%)
Inception-v3	Kaggle Test	3.14	16.37	5.90	5.11
	TCH	5.66	59.50	34.89	49.69
ResNet101	Kaggle Test	2.54	14.00	8.56	14.23
	TCH	5.35	45.45	36.92	55.76
DenseNet121	Kaggle Test	1.38	11.74	5.79	9.52
	TCH	2.88	42.15	33.49	58.79

## Data Availability

Not applicable.
